# Evaluation of Natural Language Processing for the Identification of Crohn Disease–Related Variables in Spanish Electronic Health Records: A Validation Study for the PREMONITION-CD Project

**DOI:** 10.2196/30345

**Published:** 2022-02-18

**Authors:** Carmen Montoto, Javier P Gisbert, Iván Guerra, Rocío Plaza, Ramón Pajares Villarroya, Luis Moreno Almazán, María Del Carmen López Martín, Mercedes Domínguez Antonaya, Isabel Vera Mendoza, Jesús Aparicio, Vicente Martínez, Ignacio Tagarro, Alonso Fernandez-Nistal, Lea Canales, Sebastian Menke, Fernando Gomollón, PREMONITION-CD Study Group

**Affiliations:** 1 Takeda Farmacéutica España S.A. Madrid Spain; 2 Hospital Universitario de La Princesa Madrid Spain; 3 Instituto de Investigación Sanitaria Princesa (IIS-IP) Madrid Spain; 4 Universidad Autónoma de Madrid Madrid Spain; 5 Centro de Investigación Biomédica en Red de Enfermedades Hepáticas y Digestivas (CIBEREHD) Madrid Spain; 6 Hospital Universitario de Fuenlabrada Madrid Spain; 7 Hospital Universitario Infanta Leonor Madrid Spain; 8 Hospital Universitario Infanta Sofía Madrid Spain; 9 Hospital Universitario HM Montepríncipe Madrid Spain; 10 Hospital Universitario Infanta Elena Madrid Spain; 11 Hospital Universitario Rey Juan Carlos Madrid Spain; 12 Hospital Universitario Puerta de Hierro Majadahonda Madrid Spain; 13 Department of Software and Computing System University of Alicante Alicante Spain; 14 MedSavana SL Madrid Spain; 15 Hospital Clínico Universitario Lozano Blesa Zaragoza Spain; 16 Instituto de Investigación Sanitaria Aragón (IISA) Zaragoza Spain; 17 Universidad de Zaragoza Zaragoza Spain; 18 Centro de Investigación Biomédica en Red de Enfermedades Hepáticas y Digestivas (CIBEREHD) Zaragoza Spain; 19 See Acknowledgements

**Keywords:** natural language processing, linguistic validation, artificial intelligence, electronic health records, Crohn disease, inflammatory bowel disease

## Abstract

**Background:**

The exploration of clinically relevant information in the free text of electronic health records (EHRs) holds the potential to positively impact clinical practice as well as knowledge regarding Crohn disease (CD), an inflammatory bowel disease that may affect any segment of the gastrointestinal tract. The EHRead technology, a clinical natural language processing (cNLP) system, was designed to detect and extract clinical information from narratives in the clinical notes contained in EHRs.

**Objective:**

The aim of this study is to validate the performance of the EHRead technology in identifying information of patients with CD.

**Methods:**

We used the EHRead technology to explore and extract CD-related clinical information from EHRs. To validate this tool, we compared the output of the EHRead technology with a manually curated gold standard to assess the quality of our cNLP system in detecting records containing any reference to CD and its related variables.

**Results:**

The validation metrics for the main variable (CD) were a precision of 0.88, a recall of 0.98, and an F1 score of 0.93. Regarding the secondary variables, we obtained a precision of 0.91, a recall of 0.71, and an F1 score of 0.80 for CD flare, while for the variable vedolizumab (treatment), a precision, recall, and F1 score of 0.86, 0.94, and 0.90 were obtained, respectively.

**Conclusions:**

This evaluation demonstrates the ability of the EHRead technology to identify patients with CD and their related variables from the free text of EHRs. To the best of our knowledge, this study is the first to use a cNLP system for the identification of CD in EHRs written in Spanish.

## Introduction

Crohn disease (CD) is a chronic inflammatory bowel disease (IBD) that leads to lesions in different sites along the length of the gastrointestinal tract and, occasionally, in other extraintestinal locations such as skin, eyes, joints, mouth, and the hepatobiliary system [1]. Symptoms (including abdominal pain, diarrhea, fever, and weight loss) evolve in a relapsing and remitting manner, leading to bowel damage and disability. CD is considered to be a heterogeneous disorder with a multifactorial etiology, in which genetics and environmental factors interact to manifest the disease [2]. Although most patients with CD are diagnosed with an inflammatory phenotype, about half of them do require surgeries derived from complications such as strictures, fistulas, or abscesses [3].

Over the last years, most health care institutions have moved away from paper clinical records toward electronic health records (EHRs) in which patients’ longitudinal medical information is stored [4]. Since then, large volumes of digitalized real-world clinical data have been generated at exponential rates. Although some clinical data contained in the EHRs are stored in structured fields, the majority of the relevant clinical information appears embedded in the free-text narratives written down by health professionals [5].

The area of computer science dedicated to the analysis and representation of naturally occurring texts (written or oral) [6] is called natural language processing (NLP). One of the applications of NLP focuses on the extraction of information from free text captured in EHRs and is therefore referred to as clinical NLP (cNLP). So far, cNLP systems have been successfully applied for the extraction of relevant clinical information using approaches such as regular expressions or machine learning. As a result, the quantity and quality of data captured from the EHRs have substantially increased over recent years [7]. Although incorporating information from free text into case detection through NLP techniques improves research quality [8-10], one key challenge in this process is to ensure the validity of the results by assessing the detection performance.

In this context, as part of the PREMONITION-CD observational study, we aimed to assess the performance of the cNLP system *EHRead* technology [11-15] in identifying medical records that contain mentions of CD and its related variables when compared to the detection performed by expert medical doctors. Because the manual review of free-text narratives is extremely time-consuming, valuable information routinely collected in clinical practice has largely remained unused for research purposes. Therefore, the validated automatic extraction of this information holds potential to advance our knowledge about CD and could have a positive impact in the management of these patients [16,17].

## Methods

### Ethics Approval and Consent to Participate

This study was conducted within the scope of the PREMONITION-CD project, a multicenter, retrospective study aimed at using NLP to detect free-text information in CD patients’ EHRs. Before the start of data collection, the study was approved by the Spanish Ethics Committee, Agencia Española de Medicamentos y Productos Sanitarios, and the Madrid region Ethics Committee, Comité Ético de Investigación con Medicamentos Regional de la Comunidad de Madrid, with reference number IBD-5002 (May 2018). Approval from each of the hospitals participating in the study was also obtained. It was registered in *ClinicalTrials.gov* with the identifier number NCT03668249.

The study was conducted in compliance with legal and regulatory requirements and followed generally accepted research practices described in the ICH Guideline for Good Clinical Practice, the Declaration of Helsinki in its latest edition, Good Pharmacoepidemiology Practices, and applicable local regulations.

### Consent for Publication

In accordance with article 14.5 of the General Data Protection Regulation (GDPR), if obtaining consent is impossible or would involve a disproportionate effort, in particular for processing for archiving purposes in the public interest, scientific or historical research purposes, or statistical purposes, the study is subject to the conditions and safeguards referred to in Article 89.

Regarding Article 89 of the GDPR, processing in the public interest or scientific research purposes shall be subject to appropriate safeguards and will not require consent from each of the data subjects, in accordance with the GDPR, for the rights and freedoms of the data subject.

### Availability of Data and Materials

Due to the retrospective nature of the research, data analysis did not require consent from the data subjects. Therefore, supporting data is subject to strict confidentiality agreements with each participating hospital and cannot be made openly available.

### Data Source

Data were collected from 8 hospitals of the Spanish National Healthcare Network from January 1, 2014, to December 31, 2018 (except for one participating site with electronic data available from 2013 to 2017).

### Study Design

For this study, the assessed variables were CD, CD flare (a crucial variable for the characterization of the evolution of the disease), and vedolizumab (a biologic drug indicated exclusively for the treatment of IBD). The variables included in this study were selected by the senior study committee based on the PREMONITION-CD overall study objectives. The variables were detected when written directly in the EHRs, without inferences or prior outcome definitions. The human annotations served the purpose of the creation of a gold standard to which the EHRead technology was compared.

**Figure 1:**
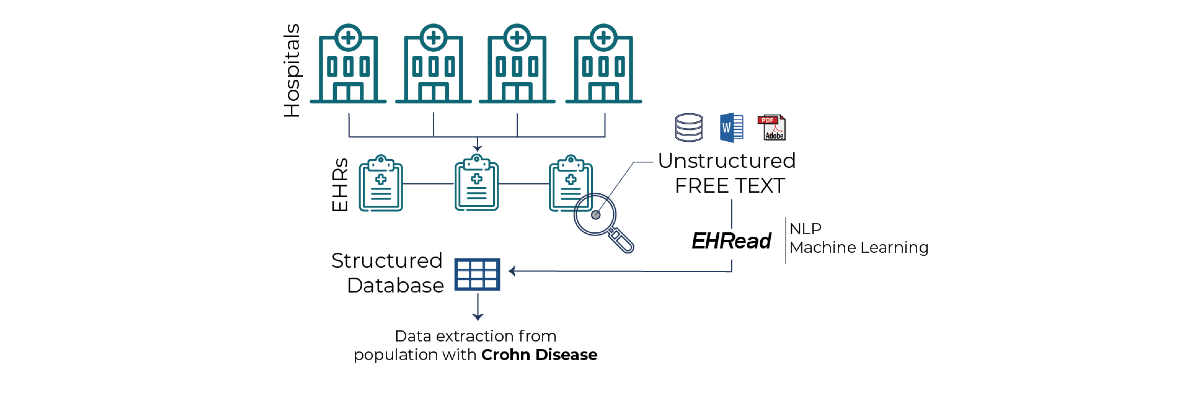
Extracting and organizing unstructured clinical data into a structured database. The EHRead technology is a clinical NLP system that detects and extracts clinically relevant information contained in deidentified EHRs. The extracted information from participating sites is organized in a structured study database. From this database, patients that fulfill the study criteria based on the study inclusion and exclusion criteria make up the target population. In this case, clinical data from the population with a diagnosis of Crohn disease were used. EHR: electronic health record; NLP: natural language processing.

The EHRead technology is an NLP system designed to retrieve large amounts of biomedical information contained in EHRs [11-15] and convert the information into a structured representation (Figure 1).

**Figure 2:**
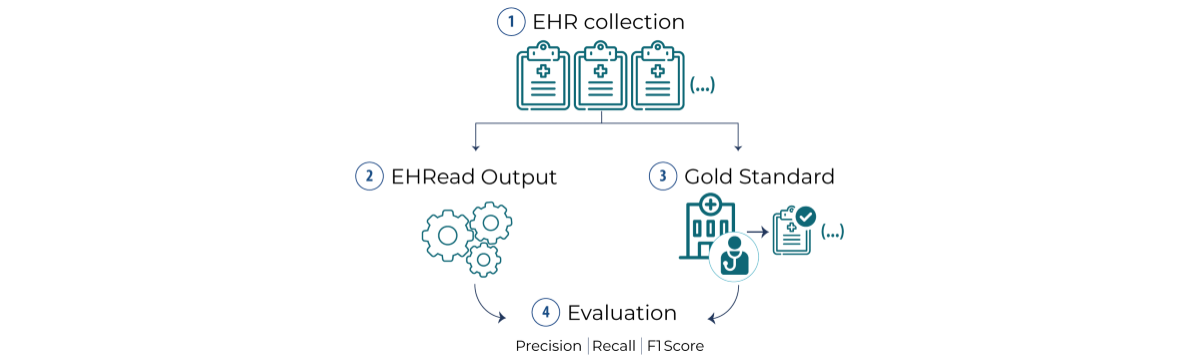
Linguistic evaluation process. To validate the output of the EHRead technology, a statistical comparison was performed between its output and a gold standard consisting of a subset of EHRs annotated by expert physicians. The validation metrics calculated are expressed in terms of precision, recall, and F1 score. See text for further details. EHR: electronic health record.

To perform this study, we completed the following steps: EHR collection, processing using EHRead technology, creation of the gold standard data set, and comparison of both outputs using standard measures of performance (Figure 2).

In the *EHR collection step*, a data set was selected that consisted of a sample collection of EHRs obtained primarily from the gastroenterology service (including consultation, hospitalization, and emergency reports), representing more than 3,900,000 patients. To obtain a representative data set, 100 records were randomly selected from each of the 8 sites containing EHRs with and without CD-related information, amounting to a total of 800 clinical documents from 800 patients. Subsequently, all records were fully anonymized to meet legal and ethical requirements before they were annotated by physicians (annotators) to generate a gold standard for each participating site (see sections about annotation process and gold standard).

In parallel to the annotation task carried out by physicians, the EHRead technology was applied on the free text of the same EHRs used to generate the gold standard (for more details see NLP System). By doing so, the performance of the EHRead technology could directly be compared to human performance in detection of CD and secondary variables.

In the final step of the evaluation, the performance of the EHRead technology was compared against the gold standard to validate the capacity of the technology in identifying records containing mentions of CD and its related variables. Therefore, both the detections of physicians and the EHRead technology were transformed into binaries (0 no detection, 1 detection) for each variable to calculate the performance metrics precision, recall, and F1 score using the library scikit-learn [18].

### NLP System

The main phases of the NLP system were the following:

- The section identification phase aims to detect the different parts of a clinical document, such as family medical history, physical exam, and treatment.
- The concept identification phase is when the system detects a medical concept. Specifically, the terminology considered by the EHRead technology is built upon SNOMED-CT (Systemized Nomenclature of Medicine–Clinical Terms), a leading platform of systematically organized and computer-readable collections of medical concepts. SNOMED-CT includes codes, concepts, synonyms, and definitions used in clinical documentation and is considered the most comprehensive terminology worldwide.
- The contextual information phase focuses on detecting the attributes of the already identified clinical terms within their textual context, both from an intention perspective (the term is either stated in an affirmative way or negated, or is part of a conjecture or opinion) and from a temporal perspective (current or historical).

### Annotation Process and Gold Standard

The manual revision of clinical texts was carried out by annotators specialized in gastroenterology. For the annotation task, guidelines were jointly created by internal NLP experts and clinical experts. They included the variables to be annotated in the free text, along with recommendations on how to solve uncertainties. Following these guidelines, specialists reviewed the free text of selected EHRs for the occurrence of the study variables to answer a set of yes/no questions: Does/did the patient have CD? Does the report state that the patient has had a flare? and Does the record state that the patient was treated with Vedolizumab? The second and third questions were only asked if the first one was affirmative, meaning that the patient did have CD before or at the time point of the hospital visit. The annotators were not allowed to respond with *yes* to any of the questions based on inference.

Of the 100 records selected per site, 15 were reviewed by two independent annotators to assess the interannotator agreement [19,20]. A low agreement indicates that the annotators had difficulties in linguistically identifying the relevant variables in the EHRs or that the guidelines are still inadequate in properly describing the annotation task [21]. Thus, the interannotator agreement serves as a control mechanism to check the reliability of the annotation and further to establish a target of performance for the NLP system. For this task, the annotators were not allowed to communicate with each other or share information regarding the annotation process to avoid bias. Once the annotations were finished, the interannotator agreement was calculated in terms of F1 score. Once the quality of annotations had been verified through the interannotator agreement and the disagreements had been resolved to build the final gold standard, one of the two physicians annotated the remaining 85% of clinical records to complete the gold standard.

### Statistical Analysis

The performance of the EHRead technology in identifying CD and its related variables was compared with the gold standard. The agreement between them was calculated using three metrics: *precision* (ie, positive predictive value), *recall* (ie, sensitivity), and their harmonic mean *F1 score* [21]. *Precision* is the indicator of the accuracy of information retrieved by the system, *recall* is the indicator of the amount of information the system retrieves, and *F1 score* conveys the balance between precision and recall. In addition to those metrics, we calculated the 95% CI for each aforementioned measure, since this provides information about the range in which the true value lies and thus how robust the metric is. The method used to calculate the 95% CIs is the Clopper-Pearson approach, one of the most common methods for calculating binomial 95% CIs.

## Results

The gold standard data set (N=800) consisted of 41.4% (n=331) medical records with CD, 21.3% (n=170) with CD flare, and 10% (n=83) with vedolizumab treatment. Table 1 shows the interannotator agreement F1 scores of the gold standard for each investigated variable per site.

The interannotator agreement values were higher than 0.8 for all comparisons, indicating an *almost perfect* agreement according to the Landis and Koch scale [19]. In addition, the overall agreement between all sites was *almost perfect* [22] for the three studied variables. The EHRead technology results in terms of *precision*, *recall*, and *F1 score* are shown in Table 2.

The detection of the main variable (ie, CD) achieved a *precision* of 0.88, a *recall* of 0.98, and an *F1 score* of 0.93. Regarding the secondary variables, CD flare obtained a *precision* of 0.91, a *recall* of 0.71, and an *F1 score* of 0.80, while the variable vedolizumab was detected at a *precision* of 0.86, a *recall* of 0.94, and an *F1 score* of 0.90.

**Table 1:** Interannotator agreement (F1 score) per participating site.

	F1 score		
	Crohn disease	Crohn disease flare	Vedolizumab
Site 1	0.93	0.86	1.00
Site 2	1.00	0.87	1.00
Site 3	1.00	1.00	1.00
Site 4	0.93	1.00	1.00
Site 5	0.93	0.83	1.00
Site 6	0.93	1.00	1.00
Site 7	1.00	1.00	1.00
Site 8	1.00	0.85	1.00
Average	0.97	0.93	1.00

**Table 2:** Performance of the EHRead technology.

Variable	Precision (95% CI)	Recall (95% CI)	F1 score (95% CI)
Crohn disease	0.88 (0.85-0.91)	0.98 (0.95-0.99)	0.93 (0.90-0.95)
Crohn disease flare	0.91 (0.85-0.95)	0.71 (0.63-0.77)	0.80 (0.72-0.85)
Vedolizumab	0.86 (0.76-0.93)	0.94 (0.86-0.98)	0.90 (0.81-0.96)

## Discussion

The evaluation presented here is part of the observational, retrospective PREMONITION-CD study, designed to characterize clinical and nonclinical variables of patients with CD. To the best of our knowledge, this is the first multicentric study using a cNLP system for the identification of prespecified CD-related variables from reports written in Spanish. The intrinsic characteristics of IBD and the current dilemmas associated with the medical management of affected patients present an opportunity for the implementation of big data research strategies. Artificial intelligence techniques complement current research efforts and might be key in disentangling the complexity of the disease [23] by allowing key patient-centered information to be retrieved and analyzed at a larger population scale. In turn, large CD/IBD data sets will enable the identification of clinical patterns, patient management, and predictors of disease that will ultimately improve patient care.

Although some clinical data is stored in structured fields of EHRs, the majority is contained in the narrative free text [4]. The automated extraction of these data using modern NLP techniques has a strikingly positive impact on clinical practice, since it enables the exploration of this valuable patient information at a scale that was not possible before. Here, we evaluated Savana’s EHRead technology, a cNLP system designed to detect and extract clinically relevant information from the free text of EHRs [11-15], to identify CD reports from narrative clinical data.

In contrast to other research studies that applied NLP techniques on Spanish EHRs obtained from a single medical center [24,25], this study combined data from eight large hospitals, thereby providing robustness and enabling generalizability. The capabilities of the EHRead technology allowed us to process a wide range of document types and to handle the different internal structures of clinical reports from the different participating sites. In addition, the inclusion of different sites enhanced the variability and richness of the language regarding the evaluated variables. Indeed, the variables evaluated were expressed in different ways across sites, including discrepancies in abbreviations or acronyms.

*F1 scores* higher than 0.80 for all interannotator agreements ensure that the gold standard met the criteria to serve as reference. In addition, our study demonstrates a good performance of the EHRead technology in identifying reports that contain mentions of CD and CD-related variables. We obtained *F1 scores* higher than 90% for the main variable and close to 80% for the remaining variables (Table 2). Despite the intrinsic heterogeneity of EHRs resulting from a variability in physicians, data collection sites, and record completeness, EHRead was successful at pinpointing important information, as reflected by these assessment parameters. Indeed, *precision* and *recall* were balanced for most of the variables, showing that the EHRead technology is not only accurate when detecting the evaluated variables but also in terms of retrieving a large amount of information.

Although this study deals with EHRs in Spanish, most previous cNLP systems focused on information extraction from clinical reports in English [26]. *F1 scores* of cNLP systems that target EHRs in English range from 0.71 to 0.92 [27-31]. Available rule-based [24,31] or machine learning–oriented [25] systems that identify medical entities in Spanish have reached *F1 scores* between 0.70 and 0.90. However, the cNLP systems targeting the Spanish language are still limited. A direct comparison between the EHRead technology and these state-of-the-art approaches is complicated due to differences in gold standard creation and use of language. Nevertheless, the overall performance of the EHRead technology across the eight participating sites with the achieved *F1 scores* demonstrates that the performance is comparable to other state-of-the-art NLP systems available in the clinical domain. Furthermore, compared to previous works that detect CD-related variables in English using NLP to increase or correctly classify the number of patients with CD detected through the standard International Classification of Diseases-9 coding system [32,33], our study relies on a purely NLP-dependent detection approach. Having performed our study in Spanish is an added value, since it is a language in which NLP has not been previously applied in CD studies, nonetheless yielding robust results compared to previous approaches in English.

A robust linguistic validation of the EHRead technology sets it forth as a valuable methodology for future studies regarding IBD and CD. The expanding use of EHRs and the wealth of information contained within their free text represent a unique source of data that benefits from the development of cNLP systems. Indeed, cNLP systems are dynamic and evolve with novel technologies that improve concept identification [21]. This approach is suitable to better detect clinical information of patients with IBD and CD in a real-world setting, which can provide insight to improve the medical management of these patients.

In conclusion, this study presents an evaluation of the EHRead technology, an NLP system for the extraction of clinical information from the narrative free text contained in EHRs. This evaluation clearly demonstrates the ability of the EHRead technology to identify mentions of CD and two related variables. Although further research is needed, the use of the EHRead technology facilitates the automated large-scale analysis of CD, thus contributing to the improvement of clinical practice by generating real-world evidence. Robust data extraction and precise variable detection are key to support future studies using large data sets of patients with CD.

## Abbreviations

CD: Crohn disease
cNLP: clinical natural language processing
EHR: electronic health record
GDPR: General Data Protection Regulation
IBD: inflammatory bowel disease
NLP: natural language processing
SNOMED-CT: Systemized Nomenclature of Medicine–Clinical Terms
